# Ethical Considerations Associated with “Humanitarian Drones”: A Scoping Literature Review

**DOI:** 10.1007/s11948-021-00327-4

**Published:** 2021-08-03

**Authors:** Ning Wang, Markus Christen, Matthew Hunt

**Affiliations:** 1 Institute of Biomedical Ethics and History of Medicine, University of Zurich, Zurich, Switzerland; 2 Digital Society Initiative, University of Zurich, Zurich, Switzerland; 3 School of Physical and Occupational Therapy, McGill University, Montréal, Canada; 4 Centre for Interdisciplinary Research in Rehabilitation, Montréal, Canada

**Keywords:** Humanitarian aid, Drones, Disasters, Ethics, Humanitarian innovation

## Abstract

The use of drones (or unmanned aerial vehicles, UVAs) in humanitarian action has emerged rapidly in the last decade and continues to expand. These so-called ‘humanitarian drones’ represent the first wave of robotics applied in the humanitarian and development contexts, providing critical information through mapping of crisis-affected areas and timely delivery of aid supplies to populations in need. Alongside these emergent uses of drones in the aid sector, debates have arisen about potential risks and challenges, presenting diverse perspectives on the ethical, legal, and social implications of humanitarian drones. Guided by the methodology introduced by Arksey and O’Malley, this scoping review offers an assessment of the ethical considerations discussed in the academic and gray literature based on a screening of 1,188 articles, from which we selected and analyzed 47 articles. In particular, we used a hybrid approach of qualitative content analysis, along with quantitative landscape mapping, to inductively develop a typology of ethical considerations associated with humanitarian drones. The results yielded 11 key areas of concern: (1) minimizing harm, (2) maximizing welfare, (3) substantive justice, (4) procedural justice, (5) respect for individuals, (6) respect for communities, (7) regulatory gaps, (8) regulatory dysfunction, (9) perceptions of humanitarian aid and organizations, (10) relations between humanitarian organizations and industry, and (11) the identity of humanitarian aid providers and organizations. Our findings illuminate topics that have been the focus of extensive attention (such as minimizing risks of harm and protecting privacy), traces the evolution of this discussion over time (i.e., an initial focus on mapping drones and the distinction of humanitarian from military use, toward the ethics of cargo drones carrying healthcare supplies and samples), and points to areas that have received less consideration (e.g., whether sustainability and shared benefits will be compromised if private companies’ interest in humanitarian drones wanes once new markets open up). The review can thus help to situate and guide further analysis of drone use in humanitarian settings.

## Introduction

Globally, aid agencies widely use emerging technologies in humanitarian, development, and healthcare settings (Hunt et al., 2016; van Wynsberghe et al., 2018; Wang, 2020; Wang, 2021a). One prominent type of technology is unmanned aerial vehicles (UAVs), also known as drones,^1^ which represent the first wave of aerial robotics applied in humanitarian projects (Mesmar et al., 2016). They have been put to multiple uses across different humanitarian crises, including: damage inspection during the 2010 earthquake in Haiti, rescue logistics following Typhoon Haiyan in the Philippines in 2013, medical equipment delivery during the 2014 Ebola outbreak in West Africa, and topographic mapping in the aftermath of the 2015 Nepal earthquake.

Technological innovations in crisis response intersect with moral values, norms, and commitments, and may challenge humanitarian principles (Sandvik & Lohne, 2013, 2014; Sandvik, 2015). Hence, analysis of ethical challenges associated with humanitarian innovation, including drones, is required for understanding what is at stake. Our own research on the use of drones for humanitarian and development purposes (Wang, 2020; Wang, 2021a) indicates that ethical considerations associated with the humanitarian use of drones vary and extend beyond the “usual suspects” such as privacy, consent, and safety. In this work, we present a scoping review (Arksey & O’Malley, 2005; Levac et al., 2010) of the academic and gray literature to provide a comprehensive overview of how ethical considerations are discussed in the literature related to using drones in the humanitarian and development contexts.

We aim to inform the ongoing debate by mapping prevailing perspectives and identifying knowledge gaps with respect to ethical considerations in the humanitarian use of drones (Sandvik & Jumbert, 2016). Within this context, we are especially interested in identifying salient ethical considerations that have received less attention in the ongoing debate. More specifically, our objective is to assess how ethical considerations associated with the humanitarian use of drones are discussed in the academic and gray literature. To clarify the meaning of the “humanitarian use” of drones, we applied two criteria: (1) the use of drones is carried out through voluntary or solicited *humanitarian assistance* from the global aid sector; and (2) drones are operated by, or in collaboration with, *humanitarian organizations* to support aid provision.

## Methods

We followed the methodology introduced by Arksey and O’Malley (2005). We developed our review protocol with support from two librarians with expertise related to bioethics and engineering. Prior to the final data collection, we pilot-tested and calibrated the protocol to ensure its applicability.

### Research Question and Search Terms

The question guiding our scoping review was, “What is known about the ethical considerations associated with the humanitarian use of drones?” The three central notions in our review, therefore, are “drones,” “humanitarian use,” and “ethical considerations,” and are defined as follows:

The term “**drones**” refers to UAVs that are, in most cases, electrically powered aircraft of small size with limited flight range and duration. They fly above the ground (semi-)autonomously within or beyond a pilot’s visual line of sight (Floreano & Wood, 2015). There are various types of drones in terms of mechanical structures, such as fixed-wing, rotary-wing, and multi-copters (Christen et al., 2018). Most drones used in the humanitarian context are fixed-wing or multi-copters below 30 kg. Generally, such small drones have a number of remarkable socio-economic impacts. For instance, images collected by drones can fill a gap between expensive, weather-dependent, and low-resolution images provided by satellites, or car-based images limited to human-level perspectives and the accessibility of roads (Floreano & Wood, 2015). Thanks to their high versality and easy maneuverability, small drones have been rapidly deployed and steadily scaled up on a wide spectrum of civilian applications over the last decade (Wang, 2021a, 2021b).

By “**humanitarian use**,” we refer to the deployment of drones by humanitarian actors^2^ in three situations: (1) *acute humanitarian crisis* settings, including relief efforts during emergencies arising from events such as natural disasters, epidemic outbreaks, or mass population displacement^3^; (2) *immediate post-crisis settings*, including post-disaster recovery and reconstruction efforts for populations affected by an ongoing or recent humanitarian crisis; and (3) *long-term crisis-resilience or development settings*, including activities related to medical commodity delivery or health supply chain management after a crisis to strengthen resilience and mitigate risks. As such, we excluded both the use of surveillance drones in armed conflicts (e.g., for detecting war crimes), and other types of civilian use of drones (e.g., for recreational, journalistic, agricultural, construction, or public safety purposes) from our review. The exclusion criteria in Table 2 clarify the differences between the humanitarian use of drones and military or civilian uses.

Finally, with respect to “**ethical considerations**,” we concentrate on the ethical ramifications of drones used in the above-specified settings. We retained articles if they included implicit or explicit discussions about the humanitarian use of drones as either being consistent with, or infringing upon, moral values, responsibilities, or obligations considered important by the authors.

### Identifying Relevant Studies

Aligned with this general understanding of the three central notions, we tested different combinations of primary search terms, starting with a set of more extensive keywords. We then included secondary and tertiary search terms to assess their impact on the search results, using the approach of the systematic inclusion of single terms. Table 1 shows the resulting search strings using the “AND” function. We adapted the use of these strings to the specificities of the selected databases.

**Table 1:** Search strings used in the database searches

Central notion	Search string
Drones	Drone* OR “unmanned aerial vehicle*” OR “unmanned aerial system*” OR “UAV*” OR “UAS*”
Humanitarian use	Humanitarian* OR emergenc* OR “aid” OR “disaster” OR “rescue” OR “relief” OR “first response”
Ethical considerations	Ethic* OR “moral” OR “legal” OR regulat* OR social

We removed the terms “remotely piloted aircraft” (RPA) and “remotely-piloted aircraft system” (RPAS) from the search string based on the testing results for a number of reasons, including the fact that they mostly yielded military applications of drones, which we deliberately excluded from our review (see Criterion D in Table 2).

To keep the literature search meaningful and manageable, we designed a set of parameters to help refine the search (Gough et al., 2012). We searched for articles, books and book chapters, and conference proceedings, as well as gray literature including policy documents, reviews, blog posts, and media reports of a minimal size. We excluded abstracts related solely to conference presentations, book reviews, PhD dissertations, and brief news releases. We calibrated the exclusion criteria in Table 2 through pilot testing.

**Table 2:** Exclusion criteria for article screening

Central notion	Exclusion criteria (A-K)
Drones	A. “Drone” is mentioned, but the article is clearly out of scope (e.g., about insects, neuroscience, molecular biology, non-flying robotic systems, etc.) B. The focus is on purely technological aspects of drones and drone technology (e.g., sensors, flight control, flight planning, etc.)
Humanitarian use	C. “Humanitarian” is mentioned as a keyword, but the article itself does not discuss humanitarian responses D. The focus is on the military use of drones (including the “war on terror”) E. The focus is on the civilian use of drones, with no focus on the humanitarian context (e.g., farming, environmental damage, infrastructure surveillance, etc.) F. The focus is on regulatory issues of civilian drones, with no focus on the humanitarian context (e.g., airspace integration, standardization, etc.)
Ethical considerations	G. “Ethics” is mentioned as a keyword, but the article does not discuss ethical issues in a substantive manner H. The article discusses ethical matters on information or robotic technology in general, but not specifically in relation to drones
Technical criteria	I. No abstract is available for further assessment (relevant for the first round of screening) J. No full text is available for further assessment (relevant for the second round of screening) K. Other technical criteria (e.g., text is too short, full text is not in English, etc.)

We only included publications in English, primarily because it is the only common language in which all researchers involved are proficient. We set the search to begin in 2000 since the first use of drones for disaster relief purposes was reported in 2005 during the response to Hurricane Katrina (Greenwood et al., 2020). Further, existing literature reviews on drones, as well as our own preliminary database search, indicated that almost no papers referring to drones were published before 2000 (Christen et al., 2018).

We used a multi-stage screening strategy involving both inductive screening via search engine and associated websites, as well as deductive identification of relevant articles in academic databases. We searched three academic databases: *Google Scholar*,^4^*Scopus*,^5^ and *Web of Science*.^6^ Our pilot test pointed to the need to adapt the search strategy in *Google Scholar* due to the high volume of search results, a consequence of the fact that the search logic in *Google Scholar* is full-text and, in addition, reveals citations of relevant texts.

In order to identify gray literature, we performed an exploratory search using the *Google* search engine and targeted website searches on 31 websites of relevant humanitarian organizations. In addition, existing resources known to the authors, as well as ad hoc advice from our project partners, served as a further source to pinpoint relevant publications. Lastly, we subjected all papers included in the final dataset to snowballing (i.e., we screened the reference sections of the papers to identify additional relevant articles).

### Selection of Articles

**Fig. 1:**
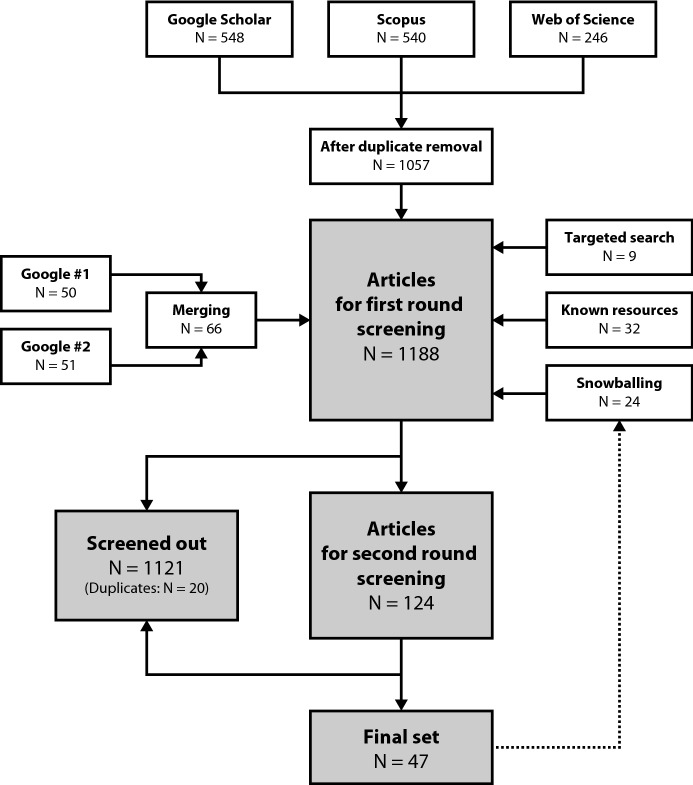
PRISMA flow chart outlining the search and selection process

We conducted the search, selection, and snowballing between April and July 2020. Figure 1 presents the process using a diagram modified from the Preferred Reporting Items for Systematic Reviews and Meta-Analyses (PRISMA) framework (Liberati et al., 2009). We included the full list of retained articles as supplementary information.

For the database searches of **academic literature** in *Scopus* and *Web of Science*, we merged the results and removed duplicates. In *Google Scholar*, we employed a refined search string that excluded the terms “conflict” and “war.” Additionally, we merged the first 100 entries in Google Scholar (sorted by “relevance”) yielded by the original search string with this set. We then merged all three sets and removed duplicates.

For the exploratory Google searches to identify **gray literature**, the first and second authors checked the first 100 search results independently (corresponding to Google #1 and Google #2 in Fig. 1). We merged and discussed findings where only one person had chosen the entry. The first author then performed targeted website searching, and the results were added to the final dataset. All three authors contributed to the inclusion of relevant sentinel articles sourced from existing knowledge (corresponding to Targeted Search and Known Resources in Fig. 1).

Using the exclusion criteria outlined in Table 2, the first and second authors independently conducted a **first round of screening** of all articles (from both the academic and gray literature) retained based on title and abstract. The aim of the screening was to classify each paper either as eligible for full-text screening, or to attribute it to one of the eight exclusion criteria. We discussed cases of conflicting assessments until reaching a consensus. In the first round of screening, the first and second author identified and removed an additional 20 duplicates.

The first author performed the **second round of screening** on the full texts of all publications that passed the first round. The first author excluded articles if they were unavailable in full-text, or if the full text was not in English, except for the abstract (see the technical exclusion criteria I, J, and K in Table 2). Finally, the first author performed snowballing on all articles included after the screenings in an iterative manner. The second author provided a second opinion whenever there was uncertainty about whether to include an article in the final set, and/or regarding the exclusion criteria.

### Charting and Analyzing the Data

We extracted data from the final selection of articles using a data extraction table, organized around the following headings:

1. *Bibliometric information*: publication date, author affiliation, and sources of articles.
2. *Contextual information*: drone use case, the type of crisis, the location of drone use, and the humanitarian organization(s) involved.
3. *Substantive information*: theories used related to ethics, and the conclusions drawn by the authors.

To identify ethical considerations, we employed a conventional **content analysis** approach whereby researchers develop inductive categorizations of the matters of concern, as opposed to applying pre-conceived notions (Hsieh & Shannon, 2005). We organized the content analysis based on an inductive, bottom-up identification of topical categories. To be comprehensive, we took an inclusive approach to interpreting “ethical considerations,” taking into account references to legal and social aspects that have a close link to ethics (as presented by the respective authors of the selected articles). To generate the categories, the first and second authors independently sketched and clustered into topics a list of descriptors taken from the text. They compared and merged the resulting classifications into a single typology. The third author then provided feedback.

### Consultation

Finally, the typology was discussed during two expert consultation workshops held on October 15, 2020. The participants included scholars with expertise in humanitarian studies, sociology, ethics, anthropology, and law, as well as practitioners from international humanitarian organizations and the drone industry. We incorporated feedback obtained from this consultation process into the revision of the typology.

## Results

**Fig. 2:**
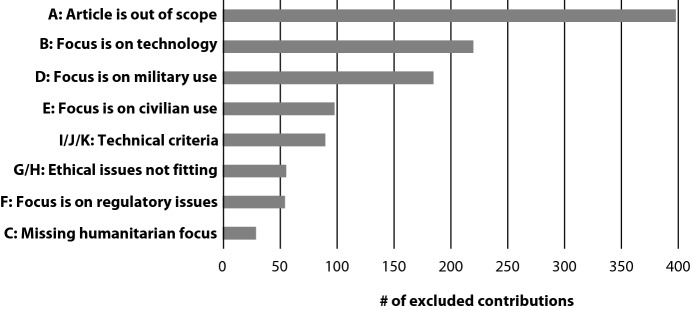
The distribution of exclusion criteria

Figure 1 above indicates that a total of 1,188 articles were subjected to the first round of screening, yielding 124 papers to be analyzed in full-text. At the end of the selection process, we retained 47 articles as relevant to our study. Figure 2 below depicts the frequency of exclusion criteria, reflecting the broader scope of our search and selection process.

### Bibliometric Information

The selected articles span from 2012 to 2020. We observed a relatively low rate of publication in the first two years with one or two articles released annually, then a steady rise between 2014 and 2018, when 26 articles were published, with an average of five articles per year. Notably, there is a significant rise in 2019 when 12 articles were published (including one article by the first author), reaching the peak of knowledge production in the review period. In 2020, five articles had already been published (including one related to the COVID-19 pandemic) by the cut-off date of our database search on April 24th. This trend of a growing discussion around the ethics of humanitarian drones indicates an expanding awareness of ethics among scholars and practitioners working in the field, echoing the rise of the so-called “good drone” in the aid sector in recent years (Sandvik & Jumbert, 2016).

While authors from six continents are represented in our dataset, a high concentration of knowledge production is seen in Europe and North America, with 21 and 18 articles published from each region, respectively. The remainder includes four from Asia (China, India, Malaysia, Singapore), two from Africa (Madagascar, Malawi), and one each from Oceania (Australia) and South America (Brazil). Among the 21 articles from Europe, the first authors of seven articles are based in Switzerland, all from outside academia; the first authors of five articles are based in Norway, all affiliated with the same research institution. Of the 18 articles from North America, three are from Canada and 15 from the US, with the first authors of nine articles affiliated with academia and six with non-academic organizations. While the majority of our dataset comprises scholarly articles produced by authors affiliated with academic institutions, the authors of 16 articles were affiliated with organizations based in the UK, the US, Canada, Switzerland, and Malawi, including three United Nations (UN) organizations (International Civil Aviation Organization [ICAO], the UN Office for the Coordination of Humanitarian Affairs [OCHA], the UN Children’s Emergency Fund [UNICEF]), one governmental organization (US Agency for International Development [USAID]), four non-governmental organizations (NGOs) (Swiss Foundation for Mine Action [FSD], FHI 360, WeRobotics, Sentinel Project), and three policy think tanks or similar organizations (Conflict Dynamics International, New America, Trilateral Research).

The collected articles were published in journals linked to six areas of study and practice: 16 articles from humanitarian/development/aid, nine from international affairs/public policy, eight from medicine/public-health, seven from engineering, five from ethics, and two from aviation. While the articles were predominantly published in social sciences and humanities journals, amounting to 30 in total, technical areas (ranging from engineering and aviation to medicine) are also important disciplinary areas. Additionally, five articles are from ethics-oriented journals, of which four are at the intersection of ethics and engineering/robotics, and one between ethics and international affairs.

### Contextual Information

Regarding the type of *drone use case*, there are 12 articles about imagery or mapping drones, ten about payload or cargo drones, six about both uses, and 19 that are unspecified. Articles referring to mapping drones were mostly produced around the period of 2014–2016, with a relatively even distribution throughout these years. In contrast, articles referring to cargo drones are mostly not seen until 2017 with a peak in 2019, of which 70% are related to healthcare or health emergencies. Before 2017, there were only four articles (one/year in 2012, 2013, 2015, and 2016, respectively) about cargo drones, and all from scholars who cautioned about the subtle dynamics between military drones and humanitarian or disaster drones, especially when used in regions previously affected by armed conflict.

In terms of *type of crisis*, 27 articles are unspecified; of the 20 articles in which a crisis can be identified, our dataset shows three main types: (1) medical emergencies, (2) healthcare, and (3) natural disasters, each representing 1/3 of the articles. One interesting use of drones in emergency situations is that of medical emergencies, such as snakebites or out-of-hospital cardiac arrest, where drones can be used to deliver anti-venom (AV) or an automated external defibrillator (AED). Moreover, epidemic or pandemic outbreaks, such as Ebola and COVID-19, present widescale medical emergencies, where drones have been deployed to facilitate relief work. However, since our primary focus is on the aid sector, which provides humanitarian relief work or development aid assistance, the medical emergency use of drones (related to AV and AEDs) is at the periphery of our research (although aid organizations do cover health emergencies during public health crises). Additionally, all seven articles about drones in natural disaster settings involve mapping drones, and all six articles about drones used in healthcare involve cargo drones.

With respect to *location of drone use*, 36 articles refer to unspecified or various locations; of the 11 articles where locations can be identified, five refer to Africa, two to the Americas, one to Europe, and one each to Asia and Australia. Interestingly, all four articles in which drones were used in the Americas and Europe were published in 2019 and 2020, and the two articles where drones were used in Oceania were published in 2017 and 2019, whereas the five articles in which drones were used in Africa were published somewhat evenly from 2014 onward. To some extent, this pattern reflects a connection between the location and timeline of drone activities; Africa has been an area of high activity for drones from the start, while Oceania, Europe, and the Americas have seen increased drone activity related to broader humanitarian use more recently.

Finally, all but 14 articles identified specific *humanitarian organizations* that used drones in different capacities, such as technical assistance or actual deployment and operations. Among the identified organizations, those mentioned more often than others were FSD, Médecins Sans Frontières (MSF, also known as Doctors Without Borders), the International Committee of the Red Cross (ICRC), OCHA, UNICEF, USAID, the World Food Programme (WFP), the World Health Organization (WHO), and the World Bank.

### Substantive Information

An overwhelming majority of the articles do not include discussions of specific theoretical approaches. Only eight articles refer to theories (including one article that mentions two theoretical approaches). Two articles are based on the “value sensitive design” (VSD) framework, and two articles show influence of science and technology studies (STS) theories, such as the “actor network theory” (ANT) and the “diffusion of innovations.” Three articles cite humanitarian principles, and two articles refer to theories of relational ethics and robot/artificial intelligence (AI) ethics. Further, these eight articles are not just from academic sources, but also from a UN organization (UNICEF) and a think tank (Conflict Dynamics International). They also represent diverse disciplines and continents, cover all use cases and crisis types, and employ different methodological approaches.

Regarding the *ethical theories* mentioned in these articles, some scholars (e.g., Cawthorne, van Wynsberghe & Comes) cited bioethics principles, including beneficence, non-maleficence, autonomy, justice, and dignity. Others (e.g., Bellievau, Meiches, Tatsidou et al.) made reference to humanitarian principles, including humanity, neutrality, impartiality, and independence. Some (e.g., Kerasidou et al., Matus & Ruytenbeek, Sandvik) also addressed specific principles, such as informed consent, do no harm, and the equitable sharing of benefits (of commercial drone use). In addition, a few scholars referred to relational ethics (Matus & Ruytenbeek), robot/AI ethics principles (van Wynsberghe & Comes), and engineering ethics principles (e.g., Cawthorne & Cenci). Overall, there is a lack of theoretical grounding of the ethical concerns discussed in most articles*.*

### Summary of Ethical Considerations

In sum, by using the conventional content analysis explained above and taking contextual and substantive information into account, we developed an initial typology inductively, which was then discussed and refined during the two expert consultation workshops. The revised typology suggests an overlap with the general ethical, legal, and social implications (ELSI) framework that is widely used for technology assessment work. In addition to the ethical considerations that emerged from the selected articles, we included legal considerations with respect mostly to regulation and governance, and social considerations, with a strong focus on the broader societal impacts of humanitarian innovation.

We acknowledge that this classification does not capture all subtleties associated with the richness and depth of ethical values such as “justice” or “respect,” and thus cannot be considered comprehensive with regard to all aspects discussed in the selected articles, as well as during the consultation workshops. Nevertheless, we consider it appropriate and sufficient to map out where the relevant issues lie. The first and second author independently identified and evaluated the tertiary-level focuses. We discussed cases with conflicting classifications until reaching a consensus.

**Fig. 3:**
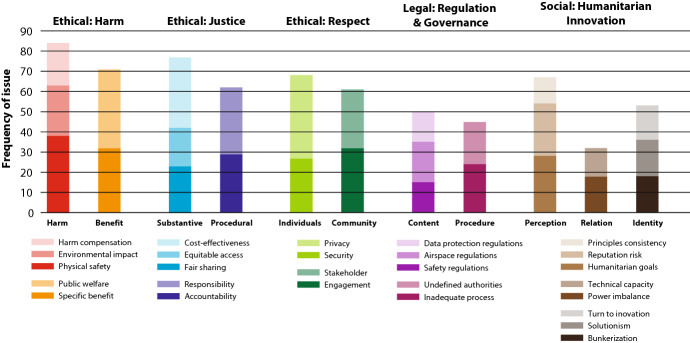
The distribution of ethical, legal, and social considerations

A more detailed analysis of the ethical considerations, outlined in Table 3, reveals the following major trends (indicated in Fig. 3). Overall, regarding **ethical considerations**, “harm” seems most prominent, followed by “justice” and “respect.” Regarding “harm,” discussions center primarily on ensuring physical safety, in addition to promoting public welfare and individual benefits for affected populations. With respect to “justice,” issues tied to procedural justice are addressed less often compared to substantive justice, whereby the cost-effectiveness of drone operations and stakeholders’ general responsibility are stressed. Concerning “respect,” the community aspect is mentioned in relation to both acceptance and engagement, while the individual aspect sheds light on privacy and information security. As for **legal considerations**, the lack of airspace regulations appears to be a concern, alongside ambiguous or inadequate regulatory processes (e.g., bureaucracy hindering drone use). Finally, in terms of **social considerations**, public perception seems to be notably represented in the literature, alongside relations between humanitarian organizations and the drone industry, as well as the identity of humanitarian aid providers and aid organizations. In particular, issues linked to the effectiveness and accountability of humanitarian aid, and the reputational risks of the military origins of drones, appear to be causing the most concern.

**Table 3:**
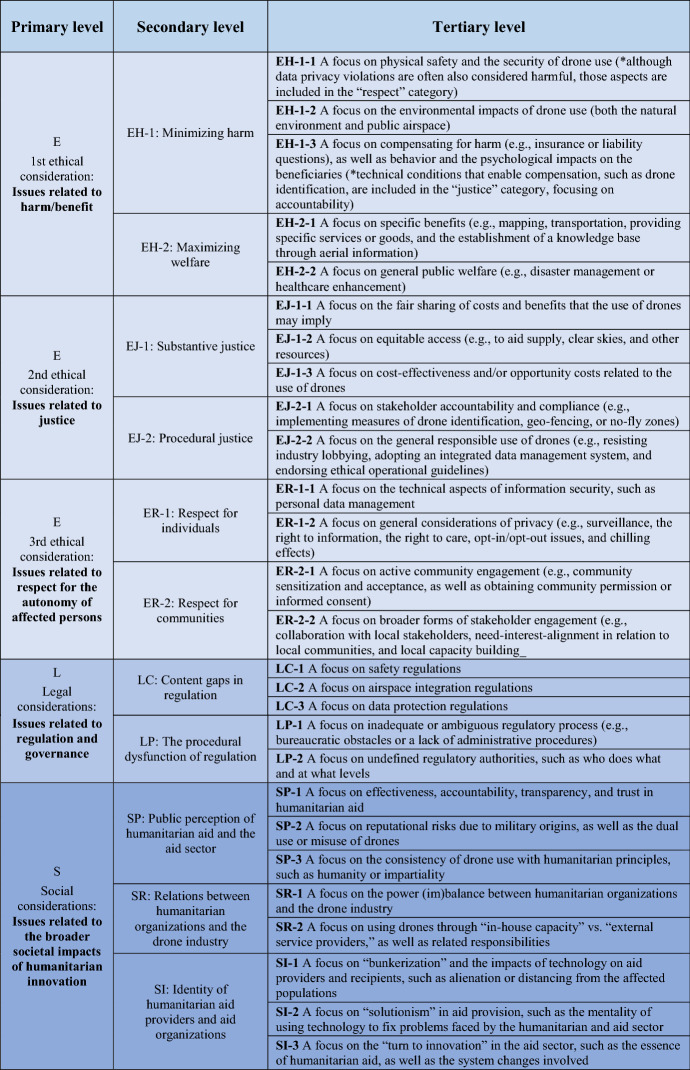
ELSI classification for analyzing the humanitarian use of drones

## Discussion

The growing trend of increased publications on humanitarian drone use and ethics reflects the increased emphasis on humanitarian innovation (Sandvik et al., 2017; Scott-Smith, 2016) and its ethical implications (Betts & Bloom, 2014; Sheather et al., 2016), as well as the broader context of rising use of drone technology across diverse sectors (Eichleay et al., 2016; OCHA, 2014; Soesilo et al., 2016; Wang, 2019, 2020, 2021a, 2021b). The bibliometric analysis of the collected articles indicated a strong growth in articles published across the review’s timespan, with the greatest number of articles released in 2019, the last complete year included. Based on this trend, and as suggested by publications early in 2020, we anticipate that this trend will continue, especially as interest in drone use appears to be strong in the humanitarian sector (Foundation for Responsible Martins, Lavallée, & Silkoset 2020; Knoblauch et al., 2019; Robotics, 2018; Tatsidou et al., 2019; USAID, 2017a, 2017b; Wang, 2020, 2021a).

A further feature that may propel this trend is that evolving regulatory environments in many countries are becoming increasingly receptive to drone use (Mauluka, 2019; Washington, 2018). Regulatory approaches to drones are also likely to have contributed to another finding from the review: where the location of drone use was specified in the articles, it was most often located in Africa. Although the number of articles identifying a specific location is relatively small, this finding is suggestive of the unfurling regulatory dynamics between regions. Compared to more recent and gradual relaxation of the regulatory atmosphere in some settings in Europe and North America, many African countries have presented more regulatory openness for drone use, as well as the development of initiatives, such as a drone testing corridor in Malawi in collaboration with UNICEF, facilitating logistical arrangements for other organizations for flight testing in a safe environment (UNICEF, 2017). This reality points to an additional concern related to the use of drones in humanitarian settings: They may offer an ideal testing environment for commercial technologies that would not be possible in other settings, and which will make possible their deployment elsewhere in non-emergency and more lucrative markets (Sandvik et al., 2017). An open question remains about whether the interest of private companies in humanitarian drones will wane once these new markets open up, with implications for the sustainability of initiatives and the shared benefits of these programs.

As reflected in our review, the area of focus for discussing humanitarian drone use has also evolved. At the start of our review period, more discussion was occurring around the use of mapping drones, a technology that was beginning to be more widely applied and commercialized in the early 2010s. In the middle period of our review, discussion of cargo drones became more prominent. Even within discourse on cargo drones, there was a shift of emphasis over time: The focus moved from ethical concerns over the intersection of military and humanitarian drone usage to a focus on the implications (e.g., privacy, risk of harm) of using cargo drones for healthcare applications, such as delivering medical supplies or transporting biological samples. The latter development is linked to the emergence of the notion and regime of the “good drone.” Several scholars of humanitarian technologies have critically appraised the good drone paradigm (Choi-Fitzpatrick, 2014; Raymond, 2012; Sandvik, 2015), who have expressed concerns associated with this shift, including the underlying motivations that have propelled it forward, and especially concerns for what this reframing might obscure, including the commercial, public surveillance, and perhaps military implications of humanitarian drone technology development. It is possible that the ethical considerations of cargo drone use will evolve over time if there is a shift away from humanitarian organizations partnering with small companies to develop humanitarian drone delivery programs, and toward the use of commercial drone delivery as these services become available in different locales. While similar technologies might be used, such a transition would reshape issues related to data management and security, control, and responsibility.

With the increasing output of articles on this topic, authorship of these sources has remained primarily with individuals based in Europe and North America, and who are commonly affiliated with academia, large international NGOs and, to a lesser extent, think tanks. This distribution is likely broadly reflective of authorship in the realm of humanitarian innovation, and humanitarian action more generally. For example, in their review of research related to disasters in low- and middle-income countries, Roy, Thakkar and Shah found that over 75% of the authors of these papers were from high-income nations (2011). Authorship patterns and publication sources also show the cross-disciplinary nature of this topic. Publications appear in a wide range of venues, such as academic journals in fields like humanitarian studies, engineering, healthcare, and ethics. Also consistent with these intersections, and with broader interest in ELSI related to emergent technologies, there appears to be particular engagement with this topic from social scientists and ethicists working in a range of domains, including STS, engineering and science ethics, and humanitarian studies. Interdisciplinary perspectives provide novel insights into the debate; for instance, van Wynsberghe and Comes (2020) proposed that analysis based on humanitarian principles (mostly answering questions about aid provision) should be complemented by a technology-oriented approach (namely robot ethics) to enrich discussions on humanitarian drone ethics. However, we noted a relative paucity of the use of theories to guide exploration of ethical considerations related to humanitarian drones. In future research on this topic, closer engagement with diverse theoretical frameworks and approaches could help to enrich the ethical investigation of these technologies (Sherman, 1999).

Through an inductive process, we identified a set of ethical considerations related to humanitarian drone use, with three main areas of emphasis: (1) optimizing harm-benefit trade-offs, (2) upholding justice, and (3) respecting autonomy. In addition, we identified considerations tied to internal and external perceptions of humanitarians and humanitarian action, as well as for regulatory and legal aspects of drone use. Broadly, the three main ethical considerations that we identified reflect core ethical concerns pinpointed in spheres such as research ethics (Belmont, 1979). Each of the three ethical considerations has two or three dimensions, which allowed us to further clarify areas of focus in the literature. For example, under the category of justice, we distinguished substantive issues of distributive and social justice from concerns related to procedural justice, such as transparency and accountability in decision-making. Across all these categories, there is a strong focus on community-level considerations, as well as for individuals. Such distinctions are reflected, for example, in discussions on harms and benefits, where either may accrue at an individual level (e.g., privacy concerns) or at a collective level (e.g., the shared benefits of mapping a landslide area). These two levels are most prominent in relation to the demonstration of respect toward individuals and communities. Respect for individuals may manifest in practices such as seeking a person’s consent, whereas community engagement activities indicate respect for the broader group of people affected by the use of a drone in a particular locality.

It is interesting to consider our typology of ethical considerations in relation to articulations of principles for humanitarian innovation. An influential example of humanitarian innovation principles is those that were developed during a joint Humanitarian Innovation Project and World Humanitarian Summit (HIP-WHS) Oxford Workshop in 2015. The principles include: being guided by a humanitarian purpose, being committed to non-maleficence (do no harm), justice (in terms of equity and fairness regarding benefits, costs and risks), accountability, the provider/recipient relationship being the primary relationship of concern, upholding autonomy (expressed as promoting the rights, dignity, and capabilities of the recipient population), and experimentation (i.e., that piloting and trials be carried out in line with international research ethics standards). These seven principles correspond well with the ethical considerations identified in our review. Concerns for maximizing benefit and minimizing risk are reflected in the emphasis on humanitarian purpose and a “do no harm” approach. The Oxford principles include both justice as a substantive concern for the distribution of benefits, risks, and harm, as well as the procedural justice concern for accountability. Likewise, respect is stressed in terms of highlighting relationships between providers and recipients of assistance, and the expectation that all innovations be aimed at advancing the rights, dignity, and capabilities of the populations affected by crises. Finally, attention to experimentation and norms of research ethics can be linked to the legal/regulatory dimensions of drone usage in that both point to questions of due oversight and structures of governance.

Across the collected articles, legal considerations mostly emerged in relation to the regulatory and governance aspects, including a lack of specific types of regulation (most prominently concerning safety and airspace management), or inadequate processes that made the use of humanitarian drones less effective (e.g., due to bureaucratic hurdles). This points to a certain ambivalence with respect to the oft repeated claim that less strict drone regulation can be an advantage for promoting drone use in crisis settings. The collected articles suggest that a lack of legislation can also create uncertainty and a perceived risk of arbitrary decisions on the part of local authorities.

The final component of our classification structure relates to perceptions of humanitarian actors. This includes both concern for perceptions of humanitarians from the perspective of the communities they aim to serve, most starkly when there is a concern that associations with the military use of drones may lead to confusion about the roles and goals of humanitarian actors. This risk also applies beyond armed conflict settings to the broader uses of drones for counter-terrorism purposes (Eckenwiler et al., 2015). In these ways, concerns may arise around credibility, security, and access, as well as perceptions of neutrality. The review also points to the ways that technology influences the relationship between humanitarian providers and populations affected by crises, and how this could lead to technological distancing between them. It is instructive to note that the HIP-WHS Oxford principles (2015) cited earlier specifically emphasize the importance of user-driven and participatory approaches for humanitarian innovation. These approaches are also important in settings where drones are being introduced, potentially guarding against both of these concerns (Wang, 2020, 2021a). Moreover, participatory approaches may be very valuable when developing new ethics guidelines for humanitarian drone use, including engaging diverse stakeholders involved in and affected by these activities (Wang et. al., forthcoming).

## Limitations

The rigor of the review was supported by steps including consultation with academic librarians, refinements to the protocol based on pilot searches, blinded searching and selection of articles by two reviewers, and two expert consultation workshops to receive feedback on provisional findings. We also acknowledge several limitations associated with this review. First, it was challenging to create boundary definitions for the concept of “humanitarian use” and to operationalize this concept in our search and selection process. We adopted a more inclusive approach to this concept by including healthcare uses of drones in low-resource health system contexts. Second, regarding our search for the concept of “ethical considerations,” we used broad terms related to ethics and morality. As a result, we may not have identified papers focused on specific ethical considerations (e.g., issues of justice) if they were not indexed in relation to these broader categories; while our search identified considerations tied to regulations and perceptions of humanitarian action, texts centered on legal or social considerations without explicitly addressing ethics would not have been identified through our search strategy. The third limitation relates to carrying out a comprehensive review of gray literature sources. This is particularly challenging in the humanitarian sector given the extensiveness of gray literature in this domain. We identified and conducted targeted searches of 31 organizational websites; we also carried out general web searches, but it is likely that we failed to identify some relevant gray literature sources through this process. The final limitation is that we restricted our search to sources written in English. While over 90% of the articles pinpointed during the pilot search were in English, it is likely that additional relevant articles were published in other languages, but were not identified based on this search parameter.

## Conclusion

“Humanitarian drones” have been increasingly used to support relief and reconstruction efforts in situations of disasters, epidemics, and population displacement, or to overcome structural barriers to healthcare delivery in low-resource settings. This scoping review presents a portrait of the expanding literature from 2012 through early 2020 related to the humanitarian use of drones, and how ethical considerations are understood and conceptualized across academic and gray literature sources. While pointing to key areas of ethical discussion related to humanitarian drone use, our review also shows that there are competing visions for the ethical implications of humanitarian drones across and within different crisis settings, and how these issues can best be addressed by different stakeholders. Our findings can also be situated within the rise of the humanitarian innovation movement, which emerged just prior to the time period of this review (HIF-ALNAP, 2019), and which has led to a growing and diverse literature in its own right, including many papers that critically examine ethical issues associated with innovative practices, processes and products, as well as efforts to develop ethics guidelines for innovation projects. Our findings shed light on what explicit and implicit ethical values are present, and how these values are being articulated and interpreted in the existing academic and gray literature. In addition to deepening understanding of ethics and humanitarian drones, our review can contribute to orienting work on the ethics of humanitarian innovation, including the development of frameworks and ethics guidelines that are value-sensitive and context-specific.

## Data Availability

Yes.

## Footnotes

1 Within the scope of this contribution, we use the terms “drones” and “unmanned aerial vehicles” (UAVs) interchangeably.

2 By “humanitarian actor,” we refer to governmental, non-governmental or private organizations, agencies and inter-agency networks that enable national or international humanitarian assistance to be channeled to locations and populations in need of relief efforts or aid supplies.

3 In our review, we regard emergencies resulting from armed conflicts, which require relief work from aid organizations, as humanitarian crises. However, we did not focus on armed conflicts themselves, during which drones may be used as weapons or as peacekeeping intervention measures. See Table 2 for exclusion criteria.

4 Google Scholar: https://scholar.google.com/.

5 Scopus (Elsevier’s abstract and citation database): https://www.scopus.com/search/form.uri?display=basic.

6 Web of Science (a citation database provided by Clarivate Analytics): https://apps.webofknowledge.com/WOS_GeneralSearch_input.do?product=WOS&search_mode=GeneralSearch.
